# A Case of Immune-Mediated Pneumonitis Associated With Dual Nivolumab and Ipilimumab Immunotherapy Treatment

**DOI:** 10.7759/cureus.40792

**Published:** 2023-06-22

**Authors:** Tala Al-Saghir, Noor Suleiman, Benjamin D Goodman, Michael W Ferguson, Sheela Tejwani

**Affiliations:** 1 Internal Medicine, Henry Ford Health System, Detroit, USA; 2 Medical Oncology, Henry Ford Health System, Detroit, USA

**Keywords:** complications, immunotherapy, metastatic melanoma treatment, immune mediated pneumonitis, yervoy, opdivo, ipilimumab, nivolumab

## Abstract

Nivolumab and ipilimumab are immunotherapy agents used in combination to treat metastatic melanoma and have proven to be efficacious. However, they have been linked to the development of immune-mediated inflammatory processes in various organ systems and tissues, including immune-mediated pneumonitis (IMP). This case report describes a 50-year-old female patient with metastatic melanoma who was treated with nivolumab and ipilimumab therapy and developed IMP as a complication. Despite treatment with steroids and infliximab, the patient's condition worsened, and she passed away due to respiratory compromise. This report emphasizes the potential for serious complications in patients receiving combination immunotherapy and highlights the importance of close monitoring and risk stratification, particularly in patients with underlying lung conditions.

## Introduction

Nivolumab and ipilimumab are immunotherapy agents used in combination to treat metastatic melanoma and have been approved by the FDA for the treatment of several other malignancies [1,2]. Ipilimumab is a monoclonal antibody that inhibits cytotoxic T-lymphocyte-associated protein 4 (CTLA-4), a T-cell activation pathway down-regulator [2,3]. Through its CTLA-4 inhibition, ipilimumab is an effective antitumor immunity upregulator and was the first agent associated with increased overall survival of patients with metastatic melanoma in a stage 3 study conducted by Hodi et al. [3]. Nivolumab is a programmed cell death (PD-1) inhibitor that, in combination with ipilimumab, has been pivotal in the treatment of advanced melanoma and untreated brain metastasis [2-4]. A study conducted by Wolchok et al. has demonstrated that in patients with advanced melanoma, the combination of nivolumab and ipilimumab resulted in significantly longer overall survival in comparison to nivolumab alone or ipilimumab alone [4].

However, the use of these agents has been associated with immune-mediated inflammatory processes in multiple organ systems and tissues, including the lungs. Multiple studies have shown that the use of the agents in combination has resulted in a higher incidence of adverse effects [2,4]. Usually, this combination immunotherapy is associated with diarrhea as a major side effect. Another complication is immune-mediated pneumonitis (IMP), which is characterized by focal or diffuse inflammation of the lung tissue and can cause respiratory symptoms such as cough, dyspnea, fever, and chest pain [5,6]. The diagnosis of IMP is made based on a combination of clinical and image-based findings [6,7]. However, these findings typically lack uniqueness and can resemble various pulmonary pathologies [7]. Therefore, the diagnosis of IMP relies on a high index of suspicion and accurately timing the onset of pulmonary symptoms in correlation with medication administration. The cessation of the immunotherapy agents or commencement of steroids with improvement is often used in these cases to confirm IMP diagnosis. 

Various clinical trials and case reports have highlighted the risk of pneumonitis associated with nivolumab and ipilimumab therapy, with some studies reporting an incidence of up to 10% in patients treated with anti-PD-1 monotherapy or combination therapy [6]. The severity of IMP can range from mild to severe, with some cases leading to respiratory failure and even death. While most outcomes have been shown to be treatable with steroids and, in severe cases, immunomodulatory agents such as infliximab, and cyclophosphamide, some rare cases can result in significant morbidity and mortality [1,6,7].

## Case presentation

Our patient was a 50-year-old Caucasian female with a past medical history of obstructive sleep apnea (OSA) on continuous positive airway pressure (CPAP), chronic obstructive pulmonary disease (COPD) on oxygen therapy of 4L at home, and stage IV malignant melanoma with metastasis to the lymph nodes and lungs on immunotherapy. She presented to our quaternary care center from an outside hospital (OSH) due to concerns about immunotherapy-induced pneumonitis, status post two rounds of nivolumab and ipilimumab.

Prior to her arrival at our center, the patient had started immunotherapy with nivolumab and ipilimumab. Seven days after her first round of immunotherapy, the patient had been hospitalized at an OSH due to worsening shortness of breath. She had been found to have a moderate left pleural effusion and dense infiltrates throughout bilateral lung fields. Cytology had been positive for metastatic melanoma, and the patient had been discharged home with a left pigtail catheter.

**Figure 1:**
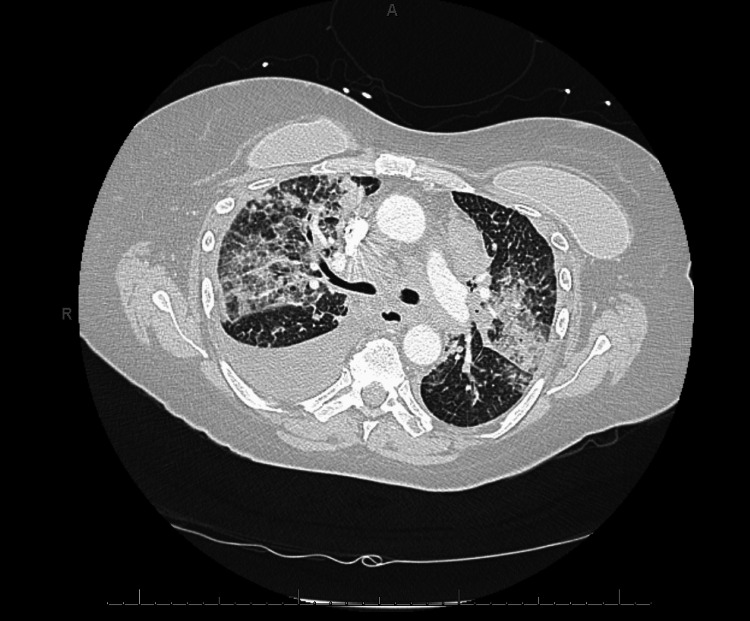
CT chest at the outside hospital CT: computed tomography

After discharge from the first OSH admission, the patient had followed up with her hematology-oncology team and undergone another round of immunotherapy, 21 days after the first round. She had subsequently developed worsening shortness of breath and her oxygen requirement had increased from 4L to 6L. Four days after her second round of immunotherapy, she had returned to the OSH. This second OSH visit had been due to worsening shortness of breath, with tachypnea, tachycardia, and hypoxia. CT imaging had shown evidence of extrinsic compression of the lower lobar bronchial tree, extensive bilateral multifocal pneumonia, and moderate right lower lobe pleural effusion (Figure 1). She had been intubated and treated with broad-spectrum antibiotics, vancomycin, and cefepime. She had also been started on methylprednisolone 40 mg every six hours and received one dose of infliximab for possible pneumonitis secondary to immunotherapy.

The patient was transferred to our center’s ICU for an interventional pulmonology evaluation and was continued on vancomycin, cefepime, and methylprednisolone. The interventional pulmonology team determined that no airway interventions were indicated. The patient was additionally evaluated by the oncology team and was found to have improved greatly on steroids, confirming that the most likely cause of her respiratory compromise was immunotherapy-mediated pneumonitis. Per the National Comprehensive Cancer Network (NCCN), the patient was classified as having “severe pneumonitis” and was continued on methylprednisolone 1 mg/kg/day with a six-week taper. Due to her clinical improvement, no further doses of infliximab were required.

On hospital day three, the patient was extubated with a 6L oxygen requirement. She was subsequently transferred out of the ICU to the general floor and was weaned off 6L to a 4L oxygen requirement. On hospital day five, the patient became hypoxic, tachypneic, tachycardic, and lethargic. She was transferred back to the ICU due to respiratory distress and started on bilevel-positive airway pressure. On day five, she decompensated, requiring re-intubation. Goals of care were established with the family and the patient was terminally weaned that same day.

## Discussion

PD-1 inhibitors, such as nivolumab, in combination with CTLA-4 inhibitor ipilimumab, have been successful in the treatment of advanced melanoma, and numerous other cancers [5]. Despite its success, the treatment can have serious health implications, particularly with regard to the rare but potentially life-threatening complication of IMP. A meta-analysis of patients on PD-1 therapy for melanoma, non-small cell lung cancer, or renal cell carcinoma treatment showed that the incidence of stage 3 or higher pneumonitis ranged from 0-4.3%, and led to pneumonitis-related death in 0.2-2.3% of patients [5]. Additionally, studies have shown that the incidence of pneumonitis is higher in patients treated with combination therapy, particularly CTLA-4 inhibitors [5,8].

Interestingly, a five-year follow-up study conducted by Larkin et al. involving patients treated with the combination therapy found that the use of the combination therapy had no difference in health-related quality of life during and after treatment compared to patients on monotherapy for advanced melanoma [1]. Additionally, the combination therapy group had higher rates of overall survival compared to patients on nivolumab combination therapy [1]. This suggests that although combination therapy has potential associated risks, the treatment benefits, minimal impact on patient quality of life, and overall positive outcomes strongly favor its use for the treatment of metastatic melanoma. Furthermore, various studies have shown that full resolution of IMP was achieved in a majority of patients post combination therapy. A study analyzing pooled data for all grades of IMP in patients with varying dosing showed IMP incidence ranging from 3.9-9% and a resolution rate of 72-94% [9]. This suggests that while this combination therapy increases the risk of developing IMP, it has a high probability of resolving. This demonstrates that while the implications of IMP are quite serious, mortality as a result of IMP is quite rare.

This is of special interest given our patient’s case where combination therapy resulted in rapid deterioration and death. The question should be raised as to whether prevailing conditions like COPD, OSA, and lung metastases predisposed this patient to a higher mortality risk in the setting of IMP. Studies have shown that in patients with fibrotic interstitial lung disease, the progression of comorbidities such as pulmonary infection, lung cancer, emphysema, and sleep disorders significantly impacted patient outcomes and resulted in increased rates of patient hospitalization [10]. Additionally, a study conducted by Naidoo et al. found that patients on monotherapy and combination therapy with underlying lung conditions had worsening clinical outcomes with IMP compared to patients with no lung conditions [8]. This indicates that our patient’s comorbidities potentially played a pivotal role in her rapid respiratory decompensation and death. The additional presence of metastasis to her lungs in the setting of IMP could have further limited her lungs' ability to successfully withstand further insults. In the same study by Naidoo et al., a majority of the patients on monotherapy and combination therapy resulting in grade 3 or higher pneumonitis achieved full recovery with drug holding/and or immunosuppression [8]. This, however, was not the case with our patient who failed to improve with proper management at our facility. These findings support the need for the consideration of multiple comorbidities in risk stratification and monitoring of patients on this combination regimen. Further studies to assess the increased incidence of IMP in patients with underlying comorbidities as well as increased mortality risk are warranted.

Finally, it is important to consider underlying comorbidities that may present similarly to IMP, masking the symptoms and hindering a medical team’s ability to identify and promptly treat complications. Our patient presented to our facility with grade 4 pneumonitis, per the Opdivo immune-mediated adverse reactions management guide [9]. Given her previous visits to the OSH after receiving the initial two rounds of immunotherapy, it is highly likely that she presented with earlier stages of pneumonitis that progressively worsened over the course of her hospital stays. While we have no way to establish it with certainty, it may explain the delay in diagnosis and treatment of the condition prior to arrival at our facility. This could have potentially worsened the IMP, leading to a poor response to the established management regimens that have been documented to be effective. While this was likely not the sole reason, this points to the importance of early screening for grade 1 and grade 2 pneumonitis and treatment withholding in patients with suspected respiratory compromise and associated comorbidities. We must emphasize the importance of establishing a baseline health assessment and particularly close monitoring for patients with relevant comorbidities to prevent complications and delayed care. We hope this case report increases awareness about this complication, particularly in the context of underlying respiratory comorbidities, to help combat undiagnosed, delayed, or untreated IMP and ultimately decrease the risk of patient morbidity and mortality.

## Conclusions

This case report highlights the potential for serious complications of IMP in patients receiving dual combination immunotherapy with nivolumab and ipilimumab, particularly in those with underlying lung conditions. Despite receiving treatment for her IMP with steroids and infliximab, our patient developed a worsening respiratory status and ultimately passed away. Her condition was possibly compounded by her comorbidities of OSA, COPD requiring home oxygen therapy, and melanoma metastasis to the lungs. The case highlights the importance of careful consideration of individual patient factors when selecting immunotherapy agents. It also stresses the need to establish a personalized baseline health assessment for each patient and vigilant monitoring and prompt intervention in individuals who develop IMP. This is particularly relevant when managing patients on dual immunotherapy, such as nivolumab and ipilimumab, due to the increased risk of immune-related adverse events in them. While recovery is possible with appropriate treatment, it should be noted that every patient's situation is unique and complications may occur, stressing the need for close monitoring and risk stratification.
